# STMS-YOLOv5: A Lightweight Algorithm for Gear Surface Defect Detection

**DOI:** 10.3390/s23135992

**Published:** 2023-06-28

**Authors:** Rui Yan, Rangyong Zhang, Jinqiang Bai, Huijuan Hao, Wenjie Guo, Xiaoyan Gu, Qi Liu

**Affiliations:** 1 Key Laboratory of Computing Power Network and Information Security, Ministry of Education, Shandong Computer Science Center (National Supercomputer Center in Jinan), Qilu University of Technology (Shandong Academy of Sciences), Jinan 250014, China; 10431210629@stu.qlu.edu.cn (R.Y.); haohj@sdas.org (H.H.); 10431210655@stu.qlu.edu.cn (W.G.); 10431210678@stu.qlu.edu.cn (X.G.); 10431210592@stu.qlu.edu.cn (Q.L.); 2 Shandong Provincial Key Laboratory of Computer Networks, Shandong Fundamental Research Center for Computer Science, Jinan 250014, China

**Keywords:** gear defect detection, lightweight network, attention mechanism

## Abstract

Most deep-learning-based object detection algorithms exhibit low speeds and accuracy in gear surface defect detection due to their high computational costs and complex structures. To solve this problem, a lightweight model for gear surface defect detection, namely STMS-YOLOv5, is proposed in this paper. Firstly, the ShuffleNetv2 module is employed as the backbone to reduce the giga floating-point operations per second and the number of parameters. Secondly, transposed convolution upsampling is used to enhance the learning capability of the network. Thirdly, the max efficient channel attention mechanism is embedded in the neck to compensate for the accuracy loss caused by the lightweight backbone. Finally, the SIOU_Loss is adopted as the bounding box regression loss function in the prediction part to speed up the model convergence. Experiments show that STMS-YOLOv5 achieves frames per second of 130.4 and 133.5 on the gear and NEU-DET steel surface defect datasets, respectively. The number of parameters and GFLOPs are reduced by 44.4% and 50.31%, respectively, while the mAP@0.5 reaches 98.6% and 73.5%, respectively. Extensive ablation and comparative experiments validate the effectiveness and generalization capability of the model in industrial defect detection.

## 1. Introduction

Gears, which are widely used as transmission components, generally have various defects on their surfaces, caused by their complex and numerous manufacturing processes. These surface defects can seriously affect their performance and service lives. Therefore, gear surface defect detection before leaving the factory becomes particularly significant.

The detection of gear surface defects usually comprises manual inspection. However, manual detection methods are heavily dependent on skilled technicians and prone to unevenness in accuracy. Moreover, the human cost is becoming increasingly high for gear manufacturers due to the decrease in the labor force. Thus, traditional machine-vision-based detection methods have emerged to replace manual inspection. These methods usually use traditional image processing techniques, such as edge detection [1] and image filtering [2], to extract the features of the gear surface. Then, classification techniques such as support vector machines (SVM) [3] and multilayer perceptron (MLP) [4] are employed for defect classification. Nevertheless, these methods are not widely used in actual gear surface defect detection due to their slow detection speeds, poor robustness, and the poor generalization ability of the manually designed features.

With the continuous advancement of convolutional neural networks (CNN) and the steady improvements in computer performance, deep-learning-based methods are rapidly developing in the surface defect detection field. An object detection network based on deep learning can accurately and precisely identify the location and category of a surface defect. YOLOv5, as a mature algorithm in the YOLO [5] series, has superior, comprehensive performance and many application cases in the industrial defect detection field. However, there are some challenges when directly applying YOLOv5 to gear surface defect detection: the brightness, type, and size of surface defects are inconsistent, since gear surface defects are affected by various external factors during the production process, which makes it difficult to detect subtle defects. Although the detection accuracy has been improved in some cases, the number of parameters, the giga floating-point operations per second (GFLOPs), and the model complexity are relatively large, which results in a low detection speed. Although some lightweight models can effectively reduce the number of model parameters and the computational costs, they cause a loss of accuracy. The balance between accuracy and speed needs to be carefully considered.

To solve the above issues, this paper proposes a YOLOv5-based lightweight gear surface defect detection model named STMS-YOLOv5, which can effectively detect three types of gear surface defects (break, lack, and scratch). The principal contributions of this paper are as follows:

(1) The lightweight ShuffleNetv2 architecture is used as the backbone to replace the CSP architecture of YOLOv5, reducing the GFLOPs and the number of parameters. Transposed convolution upsampling is adopted to achieve semantic-level upsampling, which further reduces the parameters of the model.
(2) The max efficient channel attention (MECA) mechanism is embedded in the neck to extract the critical information adaptively and enhance the multi-scale feature fusion. In addition, the Mosaic data augmentation strategy is utilized during training to enhance the dataset and improve the efficiency of the model training.
(3) Through comparison experiments with other mainstream models, it is proven that the model in this paper is effective in detecting gear defects. The detection effect of the improved model is demonstrated on a public industrial dataset (NEU-DET) [6], which verifies the generalization ability and robustness of the model.

The rest of the paper is organized as follows. Section 2 presents related work. Section 3 outlines the architecture of the YOLOv5 network. Section 4 introduces the STMS-YOLOv5 algorithm. Section 5 analyzes and discusses the experimental results. Finally, Section 6 presents the conclusions and future work.

## 2. Related Work

In recent years, with the powerful feature extraction ability of CNNs and the ability to characterize high-dimensional data, deep-learning-based methods have gradually dominated the industrial surface defect detection field, providing a basis for accurate industrial defect detection. The deep-learning-based object detection algorithms are mainly divided into two categories: (1) two-stage object detection algorithms, e.g., R-CNN [7], Fast R-CNN [8], and Faster R-CNN [9], and (2) one-stage object detection algorithms, e.g., SSD [10], YOLOv3 [11], YOLOv4 [12], and YOLOv5. The main difference between the two types of networks is that the two-stage network first needs to generate candidate boxes that may contain defects before object detection, while the one-stage network directly utilizes the features extracted from the network to predict the locations and classes of defects at the same time.

Chen et al. [13] fused the feature pyramid network (FPN) on the basis of Faster-RCNN and introduced a new visual attention mechanism (SPAM) in the backbone network to achieve the accurate detection of weld defects. Chen et al. [14] recombined ResNet-50 and deformable convolution as the backbone feature extraction network for Faster-RCNN to improve the detection performance of the algorithm for small targets, and they achieved an mAP of 93.72% on the weld defect dataset. Hu et al. [15] adopted ResNet50 as the backbone of Faster R-CNN to improve the detection of small surface defects on PCBs. Simultaneously, the basic residual units were replaced by the ShuffleNetV2 residual units to reduce the network’s computational complexity. Chen et al. [16] introduced the optimized Gabor filter into the Faster R-CNN detection network. At the same time, a two-stage training method based on genetic algorithm and backpropagation was designed to achieve the high-precision detection of fabric surface defects. Chen et al. [17] used the deep residual network Res2Net to enhance the original backbone network and improve the feature extraction ability. The weighted feature fusion module was used to improve the detection performance of small-target welding defects. Zhang et al. [18] used EfficientNet-B0 as the feature extraction network of Mask RCNN, improved the BiFPN structure, and added a CBAM attention mechanism to the branch. Although this method improves the accuracy of steel defect detection, the number of parameters is relatively large. Furthermore, although the above two-stage detection methods improve the detection accuracy, they typically require more detection time than one-stage detection algorithms. Therefore, two-stage detection algorithms have difficulty in meeting the real-time needs of industrial detection.

In the domain of industrial defect detection, compared with two-stage target detection algorithms, one-stage target detection algorithms are more commonly used because of their higher detection speeds. Chen et al. [19] replaced Darknet-53 with DenseNet-121 as the backbone of YOLOv3 for feature extraction and used the Taguchi method for the sensitivity analysis of the hyperparameters. The tested mAP was 14.98% higher than that of the traditional YOLOv3. Qi et al. [20] used a linear bottleneck structure with an inverted residual as the backbone of YOLOv3-Tiny to efficiently extract the features of track fastener defects. Deep convolution and pointwise convolution were utilized to reduce the computational complexity of the model. Su et al. [21] combined depthwise separable convolution with ResNet-34 as the feature extraction backbone network for YOLOv3, achieving the real-time detection of metal gear cross-sectional defects. The above-mentioned methods all redesign the backbone feature extraction network of YOLOv3 to achieve the precise detection of defect types. Han et al. [22] embedded a self-attention mechanism into the backbone of Tiny-YOLOv4 and added the efficient channel attention neural network (ECA-Net) into the FPN network, which significantly reduced the complexity of the original YOLOv4 algorithm and achieved the real-time detection of insulator defects. Ma et al. [23] integrated depthwise separable convolution and dual-channel attention modules as the backbone network of YOLOv4, reducing the network size. The above method uses lightweight modules to redesign the backbone feature extraction network of YOLOv4, achieving the real-time detection of defective targets. Hu et al. [24] embedded the CBAM attention module in the backbone network of YOLOv5 and proposed a fast spatial pooling pyramid structure, SimSPPF, to speed up the operation of the model and reduce the amount of computation while improving the feature extraction capability of the model. Lan et al. [25] used the lightweight Ghost module as the backbone network of YOLOv5 and embedded the CBAM attention mechanism into the neck network to improve the detection accuracy. Lang et al. [26] introduced the MobileNetV3 module as the backbone of YOLOv5 and replaced the SPPF module with the SE attention module to reduce the number of parameters and computational complexity, thus accelerating the speed in the detection of surface defects on magnetic rings. Wu et al. [27] used Ghost Conv and Ghost Bottleneck to replace the traditional convolution and bottleneck CSP module in the backbone network of YOLOv5, to reduce the number of model parameters. Shi et al. [28] decoupled the large convolution kernels in the YOLOv5 network in channel and space and introduced a lightweight coordinate attention module, reducing the number of model parameters. Chen et al. [29] proposed a new type of industrial detection network based on the improvement of YOLOX, using the efficient channel attention (ECA) mechanism and adaptive spatial feature fusion (ASFF) in the feature extraction network. The detection accuracy of the model was improved when tested on public datasets in multiple industrial fields. Table 1 presents some previous studies similar to our approach.

Based on the above work, some researchers have improved the detection accuracy by enhancing the network performance. Nonetheless, the model parameters have also increased, making it difficult to meet the requirements of real-time detection. Some researchers have simplified the model by sacrificing the detection accuracy. To achieve a balance between detection accuracy and speed and meet the requirements of gear surface defect detection under limited hardware platform resources, a novel lightweight algorithm for the detection of surface defects on gears, known as STMS-YOLOv5, has been developed.

## 3. YOLOv5 Object Detection Algorithm

**Figure 1:**
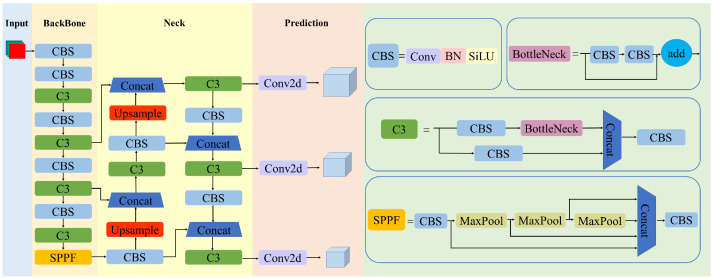
YOLOv5 network structure.

YOLOv5 is a typical one-stage object detection algorithm proposed by the Ultralytics team in June 2020, which transforms the detection task into an end-to-end regression problem. According to the depth and width of the network structure, YOLOv5 is divided into four versions, YOLOv5s, YOLOv5m, YOLOv5l, and YOLOv5x (s < m < l < x). For real-time detection and easy deployment, this paper chooses the YOLOv5s network with the smallest width and depth as the base model. The network structure of YOLOv5 is shown in Figure 1, which consists of four parts: input, backbone, neck, and prediction.

The input component of YOLOv5 consists of three modules: Mosaic data enhancement, adaptive anchor box calculation, and adaptive image scaling. Mosaic data enhancement uses four images randomly scaled, cropped, and lined up for stitching. YOLOv5 embeds the adaptive anchor box calculation function to adaptively calculate the best anchor box in different training sets. At the same time, adaptive image scaling reduces the amount of calculation and improves the inference speed. By implementing Mosaic data augmentation, adaptive image scaling, and adaptive anchor box calculation at the input stage, the accuracy and robustness of object detection have been improved.

The backbone part is mainly responsible for extracting image features at different levels of the image and consists of modules such as CBS, C3, and spatial pyramid pooling faster (SPPF). The CBS layer consists of convolution, batch normalization, and activation functions. The C3 module includes three standard convolution layers and multiple bottlenecks. SPPF uses two pooling kernels, 5 × 5 and 1 × 1, which can increase the receptive field and enter any image aspect ratio and dimension. The potential drawbacks when using the CBS, SPPF, and C3 architectures in YOLOv5’s backbone include increased computational complexity and memory usage, which lead to slower detection and increased resource requirements. Therefore, to realize the light weight of the network model, this paper adopts ShuffleNetv2 as the backbone network.

The neck feature fusion network adopts the structure of the feature pyramid network (FPN) [30] and path aggregation network (PAN) [31]. The FPN structure transfers distinctive semantic features from the top feature maps to the bottom feature maps. The PAN structure conveys robust localization features from the lower feature maps to the higher feature maps. The FPN+PAN structure used in the neck of YOLOv5 can enhance feature fusion and improve the detection accuracy by combining information from multiple scales and resolutions.

The GIOU_Loss function is used as the bounding box loss function to solve the problem of non-overlapping bounding boxes. Then, non-maximum suppression (NMS) is used to further optimize the target detection frame in the prediction stage to obtain the best size detection frame. For the GIOU_Loss function, convergence is slow in the horizontal and vertical directions when two bounding boxes intersect. Therefore, this paper chooses the SIOU_Loss function.

## 4. STMS-YOLOv5

**Figure 2:**
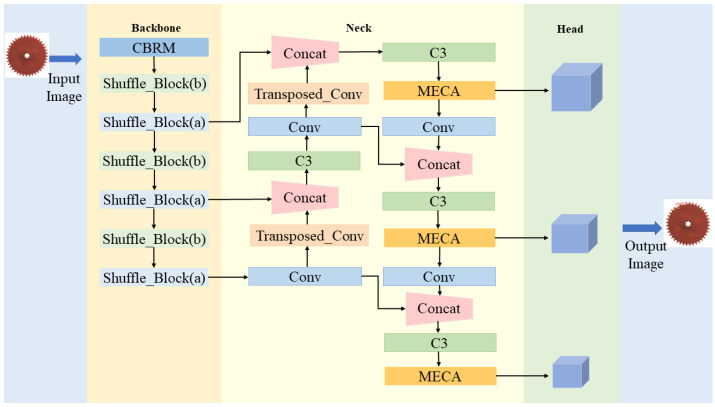
STMS-YOLOv5 network structure.

In order to balance the speed and accuracy in gear surface defect detection, this paper proposes a lightweight network named STMS-YOLOv5. Figure 2 illustrates the overall structure of STMS-YOLOv5, which comprises three parts: the backbone for feature extraction, the neck for feature fusion, and the head for location and class prediction. To reduce the number of model parameters and GFLOPs, ShuffleNetv2 is used as the backbone to extract features. To compensate for the loss of accuracy caused by the lightweight ShuffleNetv2 network, transposed convolution upsampling and MECA attention modules are embedded in the FPN and PAN of the neck part. The transposed convolution upsampling method can transfer the more distinctive features from the top feature maps to the bottom feature maps. The MECA attention modules can adaptively extract essential information. Finally, the SIOU_Loss function is used in the prediction layer to solve the problems of the GIOU_Loss function and speed up the convergence of the model.

### 4.1. ShuffleNetv2-Based Backbone

**Figure 3:**
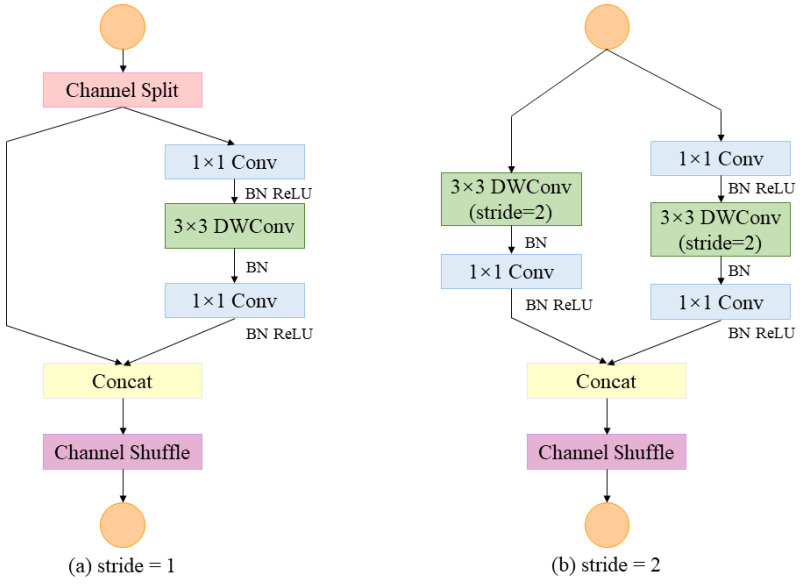
ShuffleNetv2 basic module.

ShuffleNetv2 [32] is a lightweight network proposed by Kuangshi Technology in 2018, specifically designed for embedded devices. Figure 3 shows the basic structure of ShuffleNetv2, and block (a) and (b) correspond to Shuffle_Block (a) and Shuffle_Block (b) in Figure 3. Conv is normal convolution, DWConv is depthwise convolution, BN is batch normalization, ReLU is the activation function, Channel Split refers to channel splitting, Concat refers to the splicing operation, and Channel Shuffle refers to channel shuffling.

Block (a) is a classical feature extraction module that partitions the input feature channels into two groups by channel splitting. The left branch performs constant mapping through shortcut connections to reduce fragmentation operations and speed up the model training. The right branch uses three continuous convolutions to form an inverted residual structure for feature extraction, ensuring that the input feature matrix and output feature matrix have the same number of channels, to minimize the memory access cost (MAC). Subsequently, the features from the left and right branches are spliced and shuffled through the channel shuffle operation to enable information exchange between the two branches.

Block (b) does not have the channel splitting operation but uses the DWConv with stride = 2 in both branches to realize downsampling. Then, both branches employ the 1 × 1 convolution operation to ensure that the left and right branches have the same-sized feature maps. Finally, as in block (a), the features of the left branch and right branch are concatenated and shuffled together through the concatenation and channel shuffle operation.

The backbone feature extraction network consists of the CBRM, Shuffle_Block (a), and Shuffle_Block (b) modules. The CBRM module consists of a convolutional layer, a batch normalization layer, a ReLU activation function, and a max pooling layer. The ReLU activation function easily yields large gradients in the network training process. Therefore, the ReLU activation function in CBRM is replaced by the ReLU6 activation function. Using ReLU6 can limit the output range of the activation function and enhance the stability and numerical stability of the model. The shuffle block replaces the pointwise group convolution with a channel splitting operation, which avoids an increase in the memory access cost. Moreover, this approach significantly reduces the computational complexity and the number of parameters, making it suitable for deployment on hardware-limited devices.

### 4.2. Transposed Convolution Upsampling

This paper uses transposed convolution [33] to replace YOLOv5’s nearest neighbor interpolation method for upsampling operations. Transposed convolution is a self-learning upsampling method in the image feature space. Compared with nearest neighbor interpolation, transposed convolution enables the optimization of its weights through network training, which enhances the detection accuracy. Transposed convolution is formally equivalent to the reverse gradient calculation of a convolution layer. The convolution kernel performs an inner product operation on each element of the input sequentially and finally adds up each result to obtain the output of the transpose convolution. In this way, the feature map can contain more semantic information, thereby improving the detection performance of the model.

**Figure 4:**
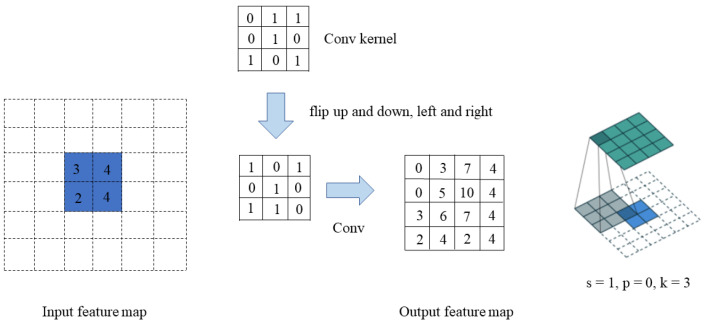
Transposed convolution.

As is shown in Figure 4, assuming that the input feature map size is 2 × 2, a feature map of size 4 × 4 is obtained after transposed convolution using a transposed convolution with a kernel size of 3, stride of 1, and padding of 0 (kernel = k, stride = s, and padding = p). First, we fill s − 1 = 0 rows and columns 0 between elements (equal to 0 without padding); then, we fill k − p − 1 = 2 rows and columns 0 around the feature map and flip the convolution kernel parameters up and down, and left and right, and finally perform normal convolution (padding 0, step 1).

Equations (1) and (2) are used to calculate the size of the feature map after the transposed convolution operation.

(1) $$H_{\mathrm{out}}=\left(H_{\mathrm{in}}-1\right)\times \mathrm{stride}\left[0\right]-2\times \mathrm{padding}\left[0\right]+\mathrm{kernel}\_\mathrm{size}\left[0\right]$$

(2) $$W_{\mathrm{out}}=\left(W_{\mathrm{in}}-1\right)\times \mathrm{stride}\left[1\right]-2\times \mathrm{padding}\left[1\right]+\mathrm{kernel}\_\mathrm{size}\left[1\right]$$

where stride [0] denotes stride in the height direction, padding [0] denotes padding in the height direction, kernel_size [0] denotes kernel_size in the height direction, and the index [1] denotes the width direction.

The transposed convolution is used to achieve upsampling because this process is learnable, and high-resolution information can be fully recovered during the parameter adjustment process. Transposed convolution can preserve more sharp boundaries than the bilinear interpolation method and enhance the fidelity of the reconstructed features.

### 4.3. MECA Attention Mechanism

In order to better extract more important gear surface defect features, this paper integrates the MECA attention module in the neck structure. The MECA attention module is a lightweight and efficient channel attention module based on the ECA [34] attention module. The attention mechanism endows the network with the capability to learn feature weights autonomously. The network can focus on important information among many inputs and effectively filter out irrelevant information, thereby addressing the potential accuracy loss problem during the lightweight phase.

**Figure 5:**
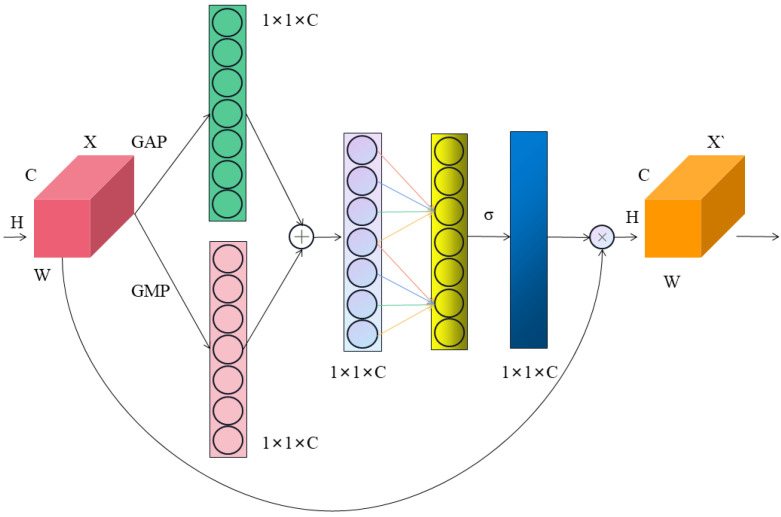
Structure of MECA attention module.

The MECA attention module is shown in Figure 5, where X represents the input feature map; *H*, *W*, and *C* represent the height, width, and channel number of the input feature map; $\tilde{X}$ represents the output feature map; GAP represents global average pooling, GMP represents global maximum pooling; σ is the activation function; and ⊗ denotes element-by-element multiplication.

Firstly, the MECA attention module passes the input feature map through global average pooling and global max pooling to obtain two 1 × 1 × *C* feature maps. Then, it adds the two feature maps to obtain a 1 × 1 × *C* feature map and uses 1 × 1 convolution to learn the channel attention information. Finally, the obtained channel attention information is multiplied with the original input feature map to obtain the final specific channel attention feature map. The MECA attention module effectively captures information from cross-channel interactions, allowing the network to locate and identify object areas more accurately.

As shown in Equation (3), there is a mapping relationship between the convolution kernel size (*k*) and the number of channels (*C*).

(3) $$k=\psi \left(C\right)={\left|\frac{\log_{2}\left(C\right)}{\gamma }+\frac{b}{\gamma }\right|}_{\mathrm{odd}}$$

Here, ${∥}_{\mathrm{odd}}$ indicates that only odd numbers can be taken, γ and *b* are set to 2 and 1, respectively, to adjust the ratio between the number of channels (*C*) and the convolution kernel size (*k*).

Global average pooling, which provides feedback for each pixel on the feature map, is utilized in the MECA attention mechanism. Global average pooling can aggregate the channel information of feature maps to achieve information sharing. The MECA attention mechanism can improve the capability of detecting objects with different scales, which reduces the rate of missed detection and compensates for the accuracy loss caused by the model’s light weight.

### 4.4. SIOU_Loss Function

The loss function of YOLOv5 consists of three parts: classification loss, bounding box loss, and confidence loss. The GIOU_Loss [35] function is used to calculate the bounding box loss.

The *GIOU*_Loss function adopts the method of first expanding the area of the union and then optimizing the *IOU* [36]. For any two bounding boxes *A* and *B*, first, we find the minimum bounding box *C* that can cover them; then, the *GIOU*_Loss function is defined by Equations (4) and (5):

(4) $$GIOU=IOU-\frac{C-(A\cup B)}{C}$$

(5) $$L_{GIOU}=1-GIOU$$

**Figure 6:**
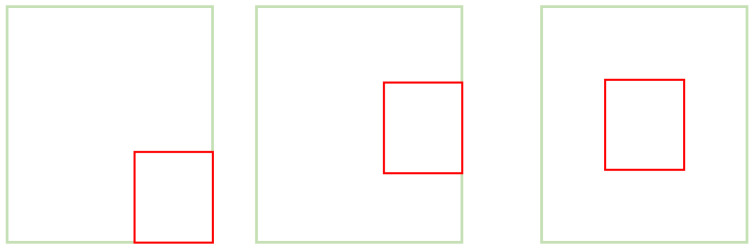
GIOU degraded to IOU.

However, as shown in Figure 6, the GIOU is degraded to the IOU when the prediction box contains the actual box. Moreover, the convergence speed is slow in the horizontal and vertical directions when the two boxes intersect. This will lead to inaccurate detection results.

To solve this issue, the SIOU_Loss [37] is used to replace the GIOU_Loss. The SIOU_Loss function redefines the distance loss by considering the vector angle between the required regressions, which can effectively reduce the degree of freedom of regression, speed up network convergence, and improve the regression accuracy.

The SIOU_Loss function consists of four cost functions, namely the angle cost, distance cost, shape cost, and IOU cost.

The angle cost allows the prediction box to move well to the nearest axis, and it is defined as

(6) $$\Lambda =1-2\sin^{2}\left(\arcsin\left(x\right)-\frac{\pi }{4}\right)$$

The distance loss is redefined based on the angle loss, and it is defined as

(7) $$\Delta =\sum_{t=x,y}\left(1-e^{-\gamma \rho_{t}}\right)$$

where $\rho_{x}={\left(\frac{b_{c_{x}}^{\mathrm{gt}}-b_{c_{x}}}{c_{w}}\right)}^{2}$, $\rho_{y}={\left(\frac{b_{c_{y}}^{\mathrm{gt}}-b_{c_{y}}}{c_{h}}\right)}^{2}$, γ=2−Λ.

The definition of the shape cost is as follows:

(8) $$\Omega =\sum_{t=w,h}{\left(1-e^{-\omega_{t}}\right)}^{\theta }$$

where $\omega_{w}=\frac{|w-w^{\mathrm{gt}}|}{\max(w,w^{\mathrm{gt}})}$, $\omega_{h}=\frac{|h-h^{\mathrm{gt}}|}{\max(h,h^{\mathrm{gt}})}$.

**Figure 7:**
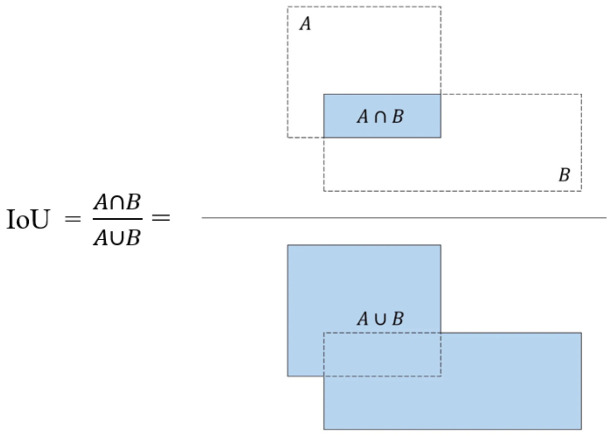
IOU calculation method.

Figure 7 shows the calculation method of the IOU loss function.

Finally, the SIOU_Loss function is defined as

(9) $$L_{SIOU}=1-IOU+\frac{\Delta +\Omega }{2}$$

SIOU_Loss introduces an angle loss factor to reduce the degree of regression freedom, which can accelerate the convergence of the network and improve the detection accuracy.

## 5. Experiments and Results

### 5.1. Experimental Environment

The hardware platform used in this experiment is as follows: Intel Xeon Processor (Icelake) CPU, 32 GB memory, Nvidia A100-SXM4 GPU, 40 GB memory. The software environment is as follows: CUDA version 11.4, Python 3.7, and PyTorch 1.10.0 as the deep learning framework.

### 5.2. Dataset and Data Pre-Processing

The gear dataset is provided by Guizhou University [38], and it has 3000 images with a size of 800 × 600 pixels and contains three defect types, i.e., break(1000), lack(1000), and scratch(1000).

The LabelImg tool [39] is employed to annotate the gear surface defect dataset. Subsequently, the dataset is divided randomly at a ratio of 8:2 to obtain the training (2400 images) and test (600 images) datasets, respectively. Then, the Mosaic9 data enhancement method is used to reduce overfitting during network training and improve the network’s generalization capability. The Mosaic9 data enhancement method randomly crops, scales, arranges, and stitches nine images into one image.

### 5.3. Model Training and Evaluation

The SGD is used as the optimizer, with a weight decay of 0.0005 and momentum of 0.937. The warm-up method is used to initialize the learning rate, and the cosine annealing algorithm is adopted to update the learning rate. The size of the input image is 640 × 640 pixels, the batch size is 128, and the initial learning rate is 0.01. The total number of training epochs is 300.

This paper uses the *mAP*, model parameters, GFLOPs, FPS, and weight size to evaluate the proposed model.

The *mAP* is calculated by the following formula:

(10) $$mAP=\frac{1}{N}\sum_{i=1}^{N}AP$$

where *N* represents n classifications and *AP* represents the average precision, which is calculated as shown in Equation (11):

(11) $$AP=\int_{0}^{1}P\left(R\right)\;dR$$

where *P* denotes precision and *R* denotes recall, which is calculated as shown in Equation (12):

(12) $$P=\frac{TP}{TP+FP}\;\;R=\frac{TP}{TP+FN}$$

True positive (*TP*) denotes the number of positive samples that are correctly predicted as positive, false positive (*FP*) represents the number of negative samples that are incorrectly predicted as positive, and false negative (*FN*) denotes the number of positive samples that are mistakenly predicted as negative.

### 5.4. Ablation Experiments

The ablation experiments are utilized to evaluate the impact of network structure changes. Five sets of ablation experiments are conducted and the results are presented in Table 2. In the ablation experiment, this paper abbreviates the ShuffleNetv2 backbone network as S, the TransposeConv upsampling as T, the MECA attention module as M, and the SIOU_Loss function as S.

As can be seen from Table 2, compared with the YOLOv5 model, the GFLOPs and parameter quantities of S-YOLOv5 are reduced by 50.92% and 47.22%, respectively, and the FPS is increased by 39.74%, which shows that ShuffleNetv2 as the backbone feature extraction network realizes a light weight and improves the detection speed. Compared with S-YOLOv5, the mAP of ST-YOLOv5 is increased by 0.2%, and the FPS is improved by 3.3, which confirms that the transposed convolution upsampling can further improve the detection speed and accuracy of the model. Compared with ST-YOLOv5, the mAP of STM-YOLOv5 is increased by 1.1%, which confirms that the MECA attention mechanism can improve the detection accuracy more efficiently. Compared with STM-YOLOv5, the mAP of STMS-YOLOv5 is improved by 0.2%, respectively. Finally, compared with the original YOLOv5 model, the GFLOPs, parameter number, and model weight of STMS-YOLOv5 are reduced by 50.31%, 44.44%, and 42.07%, respectively, while the mAP and FPS are increased by 0.8% and 40.82%, respectively. All the above analyses show that the proposed model achieves high detection accuracy as well as a fast detection speed.

**Figure 8:**
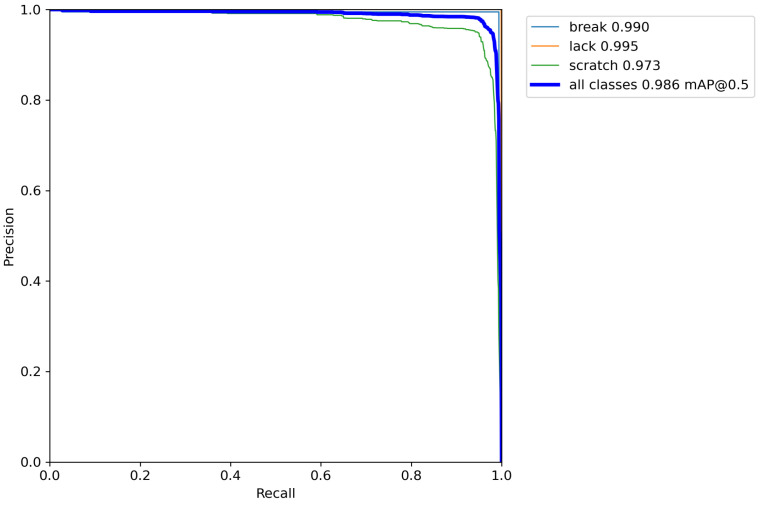
PR with IOU threshold of 0.5.

Figure 8 shows the PR curve graph when the IOU threshold is 0.5. The area between the curve and the abscissa represents the AP of the category. The closer the curve is to the upper right corner, the higher the AP value and the better the detection effect. As shown in Figure 8, the AP value of scratch is 97.3%, the AP value of break is 99.0%, and the AP value of lack is 99.5%. Thus, it shows that the proposed model can accurately identify the three types of defects on the gear surface.

To verify the generalization ability of the proposed model, the publicly available NEU-DET steel surface defect dataset is used for an ablation experiment. The NEU-DET dataset contains a total of 1800 pictures and 6 categories (crazing, inclusion, patches, pitted_surface, rolled-in_scale, and scratches). The results of the ablation experiment are shown in Table 3.

As shown in Table 3, the adoption of ShuffleNetv2 results in a 32.3 increase in the FPS but a 0.7 decrease in the mAP. The transposed convolution upsampling and MECA attention mechanism improve the mAP, compensating for the accuracy loss incurred by ShuffleNetv2. The final STMS-YOLOv5s model increases the mAP by 0.9 and the FPS by 33.3, as well as reducing the number of parameters and GFLOPs by 44.44% and 50.31%, respectively, which proves the generalization ability of the proposed model.

### 5.5. Comparison Experiments

For comparison with the ShuffleNetv2-based backbone, some lightweight networks, e.g., MobileNetv3 [40], EfficientNet [41], and GhostNet [42], are employed as the backbone of the YOLOv5s model, with the remainder of the network left unaltered. The experimental results are presented in Table 4. As shown in Table 4, when compared to other lightweight network models, ShuffleNetv2 can significantly reduce the number of parameters and model complexity without causing a significant loss of accuracy.

To verify the effectiveness of introducing the MECA attention mechanism, the MECA attention mechanism is added to the ST-YOLOv5 model and compared with the addition of the SE [43], ECA, and CBAM [44] attention modules. The experimental results are presented in Table 5, which indicates that the MECA attention mechanism achieves the maximum accuracy gain.

In order to verify the effectiveness of using the SIOU_Loss function, the SIOU_Loss function is used in the STM-YOLOv5 model and compared with the EIOU, GIOU, and CIOU loss functions. For a fair comparison, the loss value and mAP at the 249th epoch of the above loss functions were selected for comparative analysis, as shown in Table 6. The SIOU_Loss has the minimum value and achieves the maxmum mAP, which verifies the effectiveness of the improved loss function.

To evaluate the overall performance of the proposed STMS-YOLOv5 model in terms of detection accuracy, computational complexity, and model size on the gear surface defect dataset, we compare it with four other models, i.e., Faster R-CNN, YOLOv3, YOLOv4, and YOLOv5s. The results are shown in Table 7. It can be seen that the STMS-YOLOv5 model proposed in this paper is superior to the other four algorithms in terms of all evaluation indicators. The size of the STMS-YOLOv5 model is only 8.5MB, and the number of parameters is only 4.1M, but the mAP is as high as 98.6%.

**Figure 9:**
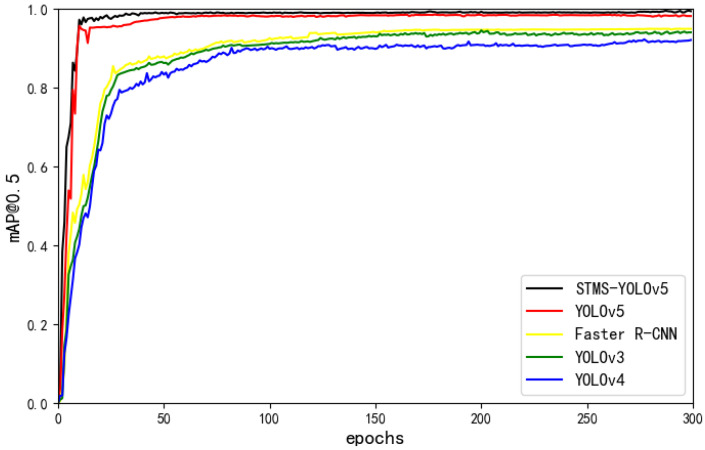
mAP with IOU threshold of 0.5.

Figure 9 shows the mAP@0.5 comparison curve of STMS-YOLOv5 and the other four models. As can be seen from the graph, STMS-YOLOv5 converges rapidly due to the adoption of the SIOU_Loss function. Although STMS-YOLOv5 has undergone lightweight processing, due to the addition of the MECA attention mechanism, the mAP does not decrease but increases slightly.

**Figure 10:**
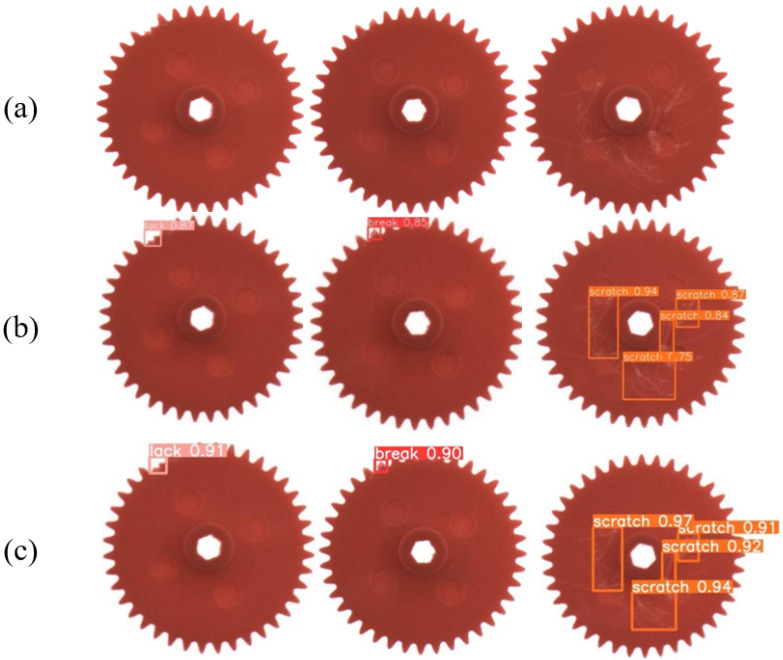
Gear detection results. (**a**) The original gear images. (**b**) The detection results of YOLOv5s. (**c**) The detection results of STMS-YOLOv5.

Figure 10 presents the detection results for three categories of gear defects on the YOLOv5s and STMS-YOLOv5 models. As shown in Figure 10, the proposed STMS-YOLOv5 model provides more comprehensive detection of defects in the gear surface, including break, lack, and scratch. The confidence scores of the detected defect categories are higher than those of the original YOLOv5s model, which fully demonstrates the excellent detection performance of STMS-YOLOv5.

**Figure 11:**
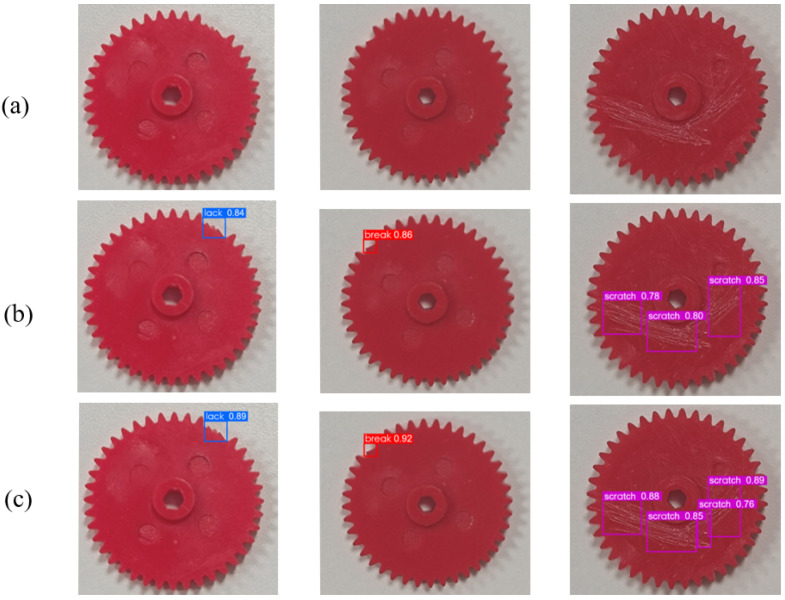
Real gear detection results. (**a**) The original gear images. (**b**) The detection results of YOLOv5s. (**c**) The detection results of STMS-YOLOv5.

To validate the performance of the proposed model on real gears, we collected 100 real gear images from the workshop. The final detection result is shown in Figure 11. STMS-YOLOv5 has better detection accuracy than the YOLOv5s model in all categories. In particular, the scratch defect can be more easily detected by STMS-YOLOv5.

## 6. Conclusions

This paper proposes a lightweight network architecture called STMS-YOLOv5 to address the trade-off between detection speed and accuracy in existing models for gear surface defect detection. The network utilizes the lightweight ShuffleNetv2 structure as the backbone for feature extraction, reducing the model complexity. Then, the network uses transposed convolution for upsampling operations to enhance the model’s learning capability. Furthermore, to compensate for the decreased accuracy caused by the lightweight backbone, the MECA attention mechanism is integrated into the neck structure. Lastly, the SIOU_Loss function is employed in the detection head section to accelerate the model’s convergence speed. The results of the ablation and comparative experiments indicate that our proposed method achieves an FPS of 130.4 and 133.5 on the gear and NEU-DET steel surface defect datasets, respectively. The number of parameters and GFLOPs are reduced by 44.4% and 50.31%, respectively, while the mAP@0.5 is improved by 0.8 and 0.9, respectively. Compared to other object detection algorithms, STMS-YOLOv5 demonstrates higher detection accuracy while ensuring a fast detection speed and controlling the model size. It effectively addresses the issue of missed detection in gear surface defect detection tasks, further confirming the strong generalization capability of the proposed algorithm in this paper.

In future research, we will expand the gear defect dataset to more comprehensively evaluate and validate the applicability of our proposed method. In terms of methods, we will explore techniques such as model pruning and knowledge distillation and continue to attempt lightweight processing to enhance the detection speed of our model.

## Figures and Tables

**Table 1:** Summary of previous studies similar to our approach.

Method	Year	Key Features
Chen et al. [19]	2021	It used DenseNet-121 as the backbone of YOLOv3 for feature extraction.
Han et al. [22]	2021	The self-attention mechanism was embedded into the backbone of Tiny-YOLOv4.
Ma et al. [23]	2022	It integrated depthwise separable convolution and dual-channel attention modules as the backbone network of YOLOv4.
Hu et al. [24]	2023	A fast spatial pooling pyramid structure (SimSPPF) was proposed to speed up the operation of YOLOv5.
Lan et al. [25]	2022	The lightweight Ghost module was used as the backbone network of YOLOv5.
Wu et al. [27]	2022	The Ghost Conv and Ghost Bottleneck was used to replace the traditional convolution and bottleneck CSP module in the network of YOLOv5.
Shi et al. [28]	2022	It decoupled the large convolution kernels in the YOLOv5 network in channel and space and introduced a lightweight coordinate attention module.
Chen et al. [29]	2023	A new type of industrial detection network based on the improvement of YOLOX.

**Table 2:** Ablation experiments on gear dataset.

Model	S	T	M	S	mAP@0.5/%	Parameters/$10^{6}$	GFLOPs/$10^{9}$	FPS	Weight Size/MB
YOLOv5					97.8	7.2	16.3	92.6	14.5
S-YOLOv5	*√*				97.1	3.8	8.0	129.4	8.0
ST-YOLOv5	√	√			97.3	3.7	8.1	132.7	8.3
STM-YOLOV5	√	√	√		98.4	4.0	8.2	130.3	8.4
STMS-YOLOv5	√	√	√	√	98.6	4.0	8.1	130.4	8.4

**Table 3:** Ablation experiment on NEU-DET dataset.

Model	mAP@0.5/%	Parameters/$10^{6}$	GFLOPs/$10^{9}$	FPS
YOLOv5s	72.6	7.2	16.3	100.2
S-YOLOv5s	71.9	3.8	8.0	132.5
ST-YOLOv5s	72.3	3.7	8.1	136.4
STM-YOLOv5s	73.2	4.0	8.2	133.5
STMS-YOLOv5s	73.5	4.0	8.1	133.5

**Table 4:** Experimental comparisons of different backbones.

Model	mAP@0.5/%	Parameters/$10^{6}$	GFLOPs/$10^{9}$	Weight Size/MB
YOLOv5s	97.8	7.2	16.3	14.5
YOLOv5s + MobileNetv3	97.3	4.3	9.4	8.7
YOLOv5s + EfficientNet	96.2	4.9	10.3	12.0
YOLOv5s + GhostNet	95.4	5.4	13.6	12.8
Ours	97.1	3.8	8.0	8.0

**Table 5:** Experimental comparisons of different attention mechanisms.

Model	mAP@0.5/%	Parameters/$10^{6}$	GFLOPs/$10^{9}$	FPS
ST-YOLOv5s	97.3	3.7	8.1	128.7
ST-YOLOv5s + SE	97.9	3.8	8.1	126.4
ST-YOLOv5s + ECA	98.0	3.8	8.1	125.8
ST-YOLOv5s + CBAM	98.2	3.9	8.2	125.2
ST-YOLOv5s + MECA	98.4	4.0	8.2	124.3

**Table 6:** Experimental comparisons of different loss functions.

Model	mAP@0.5/%	Parameters/$10^{6}$	Weight Size/MB	Loss
STM-YOLOv5s + GIOU	98.4	4.0	8.4	0.025922
STM-YOLOv5s + CIOU	98.0	4.0	8.4	0.026942
STM-YOLOv5s + EIOU	97.7	4.0	8.4	0.027605
STM-YOLOv5s + SIOU	98.6	4.0	8.4	0.024837

**Table 7:** Comparison of experimental results.

Model	mAP@0.5/%	Parameters/$10^{6}$	GFLOPs/$10^{9}$	Weight Size/MB
Faster R-CNN	94.1	60.6	180.3	108.9
YOLOv3	93.2	61.5	152.6	117.4
YOLOv4	91.5	52.5	112.4	98.7
YOLOv5s	98.2	7.2	16.3	14.5
Ours	98.6	4.1	8.1	8.5

## Data Availability

The data that support the findings of this study are available from the corresponding author upon reasonable request.
